# Management of Severe Neutropenia in a Child With Chediak-Higashi Syndrome Using Granulocyte-Colony Stimulating Factor (G-CSF): A Case Report

**DOI:** 10.7759/cureus.49010

**Published:** 2023-11-18

**Authors:** Ahmed Almesfer, Sami Alradhi, Fahad Alamr, Mohammed AlSaiary

**Affiliations:** 1 Pediatric Allergy and Immunology Unit, Maternity and Children Hospital, Dammam, SAU; 2 General Pediatric Unit, Maternity and Children Hospital, Dammam, SAU; 3 College of Medicine, Al Baha University, Al Baha, SAU; 4 Infectious Disease Unit, Maternity and Children Hospital, Dammam, SAU

**Keywords:** recurrent infections, hypopigmentation, g-csf, granulocyte-colony stimulating factor, severe neutropenia, chediak-higashi syndrome

## Abstract

Chediak-Higashi syndrome (CHS) is a congenital immunodeficiency disorder characterized by recurrent bacterial infections, oculocutaneous albinism, and abnormal intracellular protein transport. The incidence of CHS is rare, with approximately 500 cases reported so far. One of the key immunological features of CHS is neutropenia. The management of CHS includes supportive treatment, chemotherapy, methylprednisolone, IL-2 administration, and hematopoietic stem cell transplantation (HSCT). However, neutropenia can persist even after these treatments. This case report presents the successful management of severe neutropenia in an 8-year-old girl diagnosed with CHS. The patient exhibited classic CHS features, including hypopigmentation and recurrent infections. Initial treatment with antibiotics led to the resolution of the fever, but severe neutropenia persisted. Granulocyte-colony stimulating factor (G-CSF) therapy was initiated, which resulted in a substantial increase in the absolute neutrophil count (ANC) with no adverse effects. Throughout treatment with G-CSF, the patient remained stable. The patient was finally referred to the tertiary care center for consideration of bone marrow transplantation. This case highlights the potential safety and efficacy of G-CSF in managing CHS-associated neutropenia.

## Introduction

Chediak-Higashi syndrome (CHS) is a rare autosomal recessive disorder [1]. This disorder was first described by Beguez Cesar in 1943 [2]. In the 1950s, Chediak [3] and Higashi [4] further elaborated on the hematological features of CHS, highlighting the abnormal distribution of myeloperoxidases in the neutrophil granules of afflicted individuals. In recognition of their significant contribution, Sato [5] named the disease after Chediak and Higashi. The etiology of CHS is attributed to phenotype-genotype correlation. The genetic factor involved in CHS was identified in 1996 as the Lyst or CHS 1 gene [6]. This gene is involved in lysosomal trafficking and the transport of cytoplasmic granules. So far, around 40 types of mutations in this gene have been reported. These mutations in the Lyst gene disrupt protein synthesis and alter the functional capabilities of lysosomal granules, resulting in non-functional lysosomes [7]. 

The cellular characteristics of CHS include giant inclusion bodies in the cytoplasm of leukocytes due to the abnormality of vesicular trafficking. The immunological features of CHS include neutropenia and diminished natural killer cells [1]. The majority of children (85%-90%) manifest severe clinical CHS, characterized by recurrent infections and oculocutaneous albinism. A significant proportion of these children experience progression to a lymphoproliferative accelerated phase subsequent to Epstein-Barr virus exposure. The accelerated phase is usually unresponsive to chemotherapy, and bone marrow transplantation is the only viable option [8]. The management outcomes of CHS are dependent on the severity of the disease and the treatment approach used. In this paper, we present the case of an 8-year-old girl suffering from CHS who had severe neutropenia.

## Case presentation

**Figure 1:**
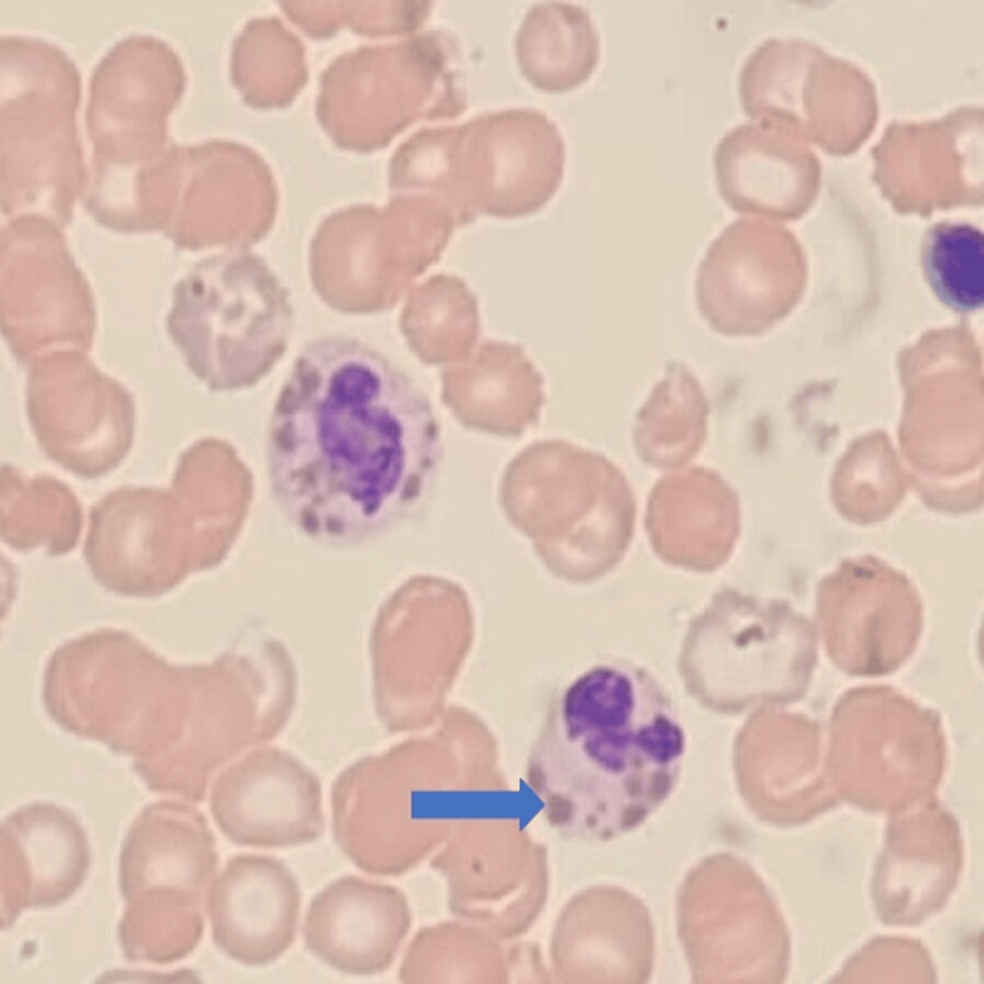
The giant abnormal granules in all granule-containing cells (blue arrow)

An 8-year-old girl was admitted to our pediatric ward with a history of prolonged fever for about 20 days, associated with a history of cough, shortness of breath, and decreased activity. The patient has a history of fair hair and abnormal pigmentation of her skin since early childhood. Additionally, she has a family history of unexplained deaths in close relatives in their early twenties. Her examination findings showed hypopigmented areas of the skin around the eyes and behind the knees. Her initial investigations (Table 1) revealed microcytic anemia, thrombocytopenia, and severe neutropenia (the absolute neutrophil count was near zero). Her peripheral blood film showed pancytopenia with no evidence of malignancy, and since her WBC count was severely affected, no giant lysosomes were seen in her blood smear. Later on, the patient underwent bone marrow (BM) aspiration and biopsy, and a blood smear was repeated. The results showed a normocellular BM with active trilineage hematopoiesis, with giant intracytoplasmic granules in all the granulocytes (Figure 1), confirming the diagnosis of Chediak-Higashi syndrome.

**Table 1:** Lab results of the patient Mean corpuscular volume, MCH: Mean corpuscular hemoglobin

Investigation	Value
Hemoglobin	7.5g/dl
MCV	61fL
MCH	22pg
Platelet	69 x 10^3^/μL
CD3	1327 (71%)
CD4	869 (47%)
CD8	369 (20%)
CD19	449 (24%)
Natural killer cells	71 (4%)

**Figure 2:**
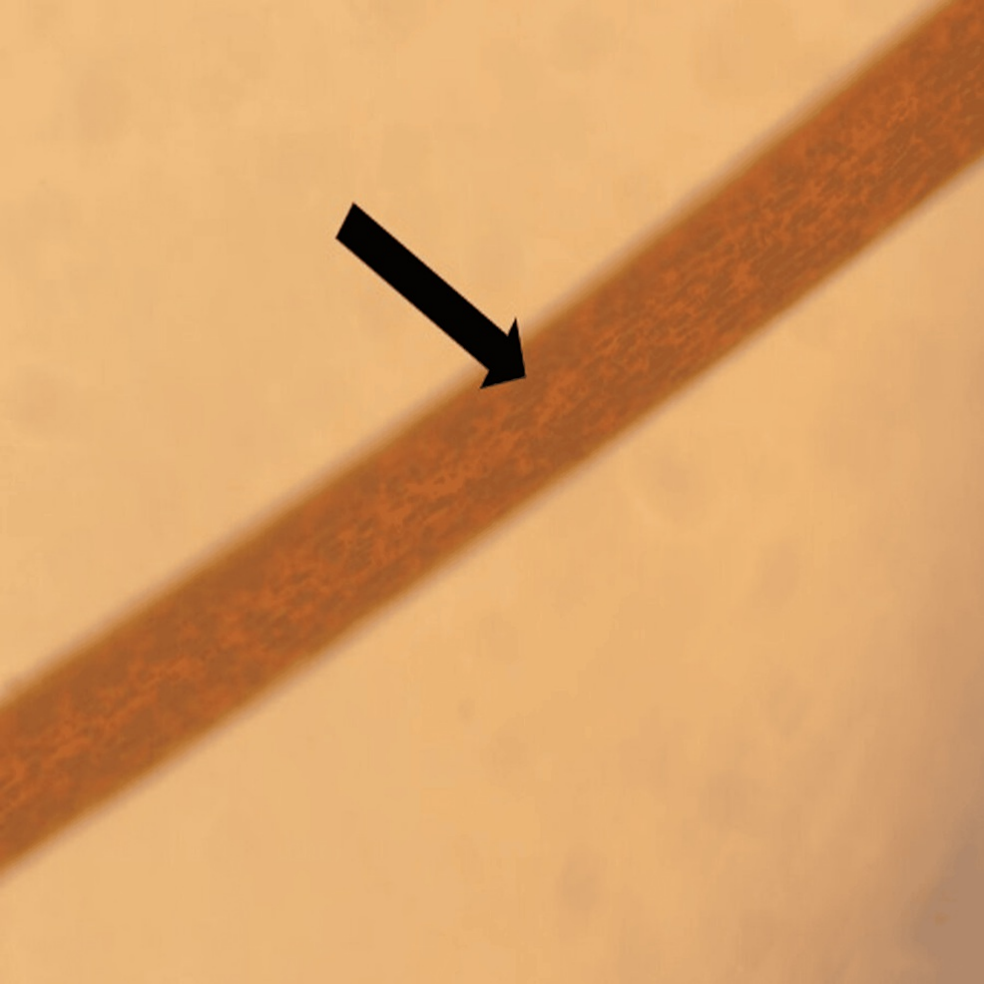
A light microscope examination of the hair shows abnormal clumps of melanin (black arrow)

A hair shaft was sent for examination under a light microscope, and the result showed regularly arranged clumps of melanin (Figure 2). The patient's abdominal ultrasound showed hepatosplenomegaly, with ferritin levels at 639.19 ug/L.

The patient was started on Piperacillin/tazobactam, and the fever subsided within a few days. However, her neutrophil count remained persistently zero. To improve the neutropenia and decrease the risk of infection, the patient was initiated on granulocyte-colony stimulating factor (G-CSF) therapy at a dose of 5mcg/kg/day for three days. Then, the therapy continued every other day, and on the 10th day, her absolute neutrophil count (ANC) increased to 7.4 x 103/μL. Throughout her hospitalization and treatment with G-CSF, the patient remained in stable condition and did not exhibit any adverse effects from the therapy. The patient was subsequently referred to a tertiary center for further evaluation and consideration of bone marrow transplantation.

## Discussion

Approximately 500 cases of CHS have been reported so far [9]. The largest study published on CHS included only 15 patients. This was a nationwide study conducted in Japan from 2000 to 2010, indicating an incidence of 1 to 2 cases per year [10]. The mean age of CHS onset is 5.85 years; however, the majority of cases die under the age of 10 years. Patients who survive beyond this period are often affected by severe neurological complications [9]. The diagnosis of CHS is usually made quite early in life. The major concern in CHS is the incidence of recurrent infections, bleeding, and progression to HLH. In the present case, the patient had a history of fair hair and abnormal pigmentation of the skin, eyes, and knees. Hypopigmentation is a common clinical symptom of CHS and is typically seen in the eyes, skin, and hair. A case series of five CHS cases by Roy et al. revealed that all patients had recurrent infections, photophobia, and silver hair [11]. Skin, mucous membrane, and respiratory tract are frequent sites of infection, with *Staphylococcus* and *Streptococcus* being the most frequently identified organisms. Bleeding and bruising in CHS patients are caused by defective platelets [12]. 

The diagnosis of CHS is confirmed during the initial stages, as the patient often has a history of recurrent infections. The clinical suspicion of CHS should be validated with a laboratory evaluation [7]. The primary or incidental diagnosis is based on the presence of giant granules in neutrophils. However, in the present case report, the neutrophil count was almost zero; therefore, a bone marrow biopsy was used for confirmation of CHS. The bone marrow aspirate from the CHS patient demonstrates active trilineage hematopoiesis, with giant intracytoplasmic granules in all the granulocytes [13]. The microscopic evaluation of hair shows clumped melanin, which is present in higher quantities compared to those observed in normal hair. Furthermore, skin examination in CHS reveals melanosomes in melanocytes and keratinocytes. The management of CHS relies on two major aspects, including supportive treatment and the alleviation of CHS-induced complications. To avoid recurrent infections in patients, hygienic practices and antibiotics are recommended [7]. 

The successful management of severe neutropenia, in this case through the application of granulocyte-colony stimulating factor (G-CSF) therapy, represents a significant achievement. G-CSF is a growth factor that promotes the maturation of the precursor cells of neutrophils, which are then transferred to peripheral blood [14]. The substantial increase observed in the patient's absolute neutrophil count following G-CSF therapy is a testament to the effectiveness of this approach. Importantly, the patient maintained a stable clinical condition throughout the course of treatment, thereby mitigating the inherent risks associated with neutropenia and its propensity for recurrent bacterial infections. Furthermore, our case report underscores the safety and well-tolerance of G-CSF therapy in a clinically stable CHS patient. While concerns persist regarding potential inflammatory responses triggered by G-CSF, our patient's experience stands as evidence to the contrary, as she did not manifest any adverse effects during the treatment regimen. This observation suggests that G-CSF therapy can be judiciously administered with a favorable safety profile in select CHS cases [15]. 

Our observation also aligns with previous case reports. For example, Shome et al. reported in their case study that the CHS patient was unresponsive to amphotericin B and packed red blood cells; however, after initiating G-CSF along with ascorbic acid, the condition of the patient improved significantly [15]. Similarly, a case of 27-year-old women with CHS presented by Baldus et al. reported that G-CSF led to the normalization of white blood cells. Furthermore, after C-GSF treatment, the patient did not experience any infection within the six-month follow-up period [16]. Other treatment options for CHS include chemotherapy, a high dosage of methylprednisolone, and IL-2 administration. Allogenic hematopoietic stem cell transplantation (HSCT) is the most successful approach before the patient enters the accelerated phase [17].

There are some limitations to this case report as well. Being a single-case report, our findings may not universally apply to all CHS patients. Larger studies involving multiple cases are needed to validate the safety and efficacy of G-CSF therapy in CHS-associated neutropenia. Additionally, the relatively short follow-up period and the patient's subsequent referral for further evaluation and potential bone marrow transplantation limit our understanding of the long-term outcomes of G-CSF therapy and its impact on the progression of CHS-associated complications. Lastly, our report does not delve into the genetic analysis of the specific CHS mutation, which could provide valuable information about disease severity and potential complications.

## Conclusions

In conclusion, this case report highlights the efficacy of C-GSF in managing neutropenia in CHS patients. Furthermore, the administration of C-GSF can also help control recurrent infections, a key clinical manifestation of CHS. After initiating C-GSF treatment, our patient remained clinically stable, which is an encouraging finding. Future studies should focus on C-GSF treatment with a larger sample size.

## References

[1] Carneiro IM, Rodrigues A, Pinho L, de Jesus Nunes-Santos C, de Barros Dorna M, Moschione Castro AP, Pastorino AC (2019). Chediak-Higashi syndrome: Lessons from a single-centre case series. Allergol Immunopathol (Madr).

[2] Baguez-Cesar A (1943). Neutoropenia cronica maligna familiar con granulaciones atipicas de los leucocitos. Bol Soc Cubbana Pediatr.

[3] CH MM (1952). [New leukocyte anomaly of constitutional and familial character]. Rev Hematol.

[4] HI O (1954). Congenital gigantism of peroxidase granules; the first case ever reported of qualitative abnormity of peroxidase. Tohoku J Exp Med.

[5] SA A (1955). Chédiak and Higashi's disease: probable identity of a new leucocytal anomaly (Chédiak) and congenital gigantism of peroxidase granules (Higashi). Tohoku J Exp Med.

[6] Nagle DL, Karim MA, Woolf EA (1996). Identification and mutation analysis of the complete gene for Chediak-Higashi syndrome. Nat Genet.

[7] Ajitkumar A, Yarrarapu SNS, Ramphul K (2018). Chediak Higashi Syndrome.

[8] Bouatay A, Hizem S, Tej A, Moatamri W, Boughamoura L, Kortas M (2014). Chediak-higashi syndrome presented as accelerated phase: case report and review of the literature. Indian J Hematol Blood Transfus.

[9] Maaloul I, Talmoudi J, Chabchoub I (2016). Chediak-Higashi syndrome presenting in accelerated phase: A case report and literature review. Hematol Oncol Stem Cell Ther.

[10] Nagai K, Ochi F, Terui K (2013). Clinical characteristics and outcomes of chédiak-Higashi syndrome: a nationwide survey of Japan. Pediatr Blood Cancer.

[11] Roy A, Kar R, Basu D, Srivani S, Badhe BA (2011). Clinico-hematological profile of Chediak-Higashi syndrome: experience from a tertiary care center in south India. Indian J Pathol Microbiol.

[12] Carnide EMG, Jacob CMA, Pastorino AC, Bellinati-Pires R, Costa MBG, Grumach AS (1998). Chediak-Higashi syndrome: presentation of seven cases. Sao Paulo Medical Journal.

[13] Sánchez-Guiu I, Antón AI, García-Barberá N (2014). Chediak-Higashi syndrome: description of two novel homozygous missense mutations causing divergent clinical phenotype. Eur J Haematol.

[14] Celkan T, Koç BŞ (2015). Approach to the patient with neutropenia in childhood. Turk Pediatri Ars.

[15] Baldus M, Zunftmeister V, Geibel-Werle G, Claus B, Mewes D, Uppenkamp M, Nebe T (1999). Chédiak-Higashi-Steinbrinck syndrome (CHS) in a 27-year-old woman--effects of G-CSF treatment. Ann Hematol.

[16] Shome DK, Al-Mukharraq H, Mahdi N, Ameen G, Farid E (2002). Clinicopathological aspects of Chediak-Higashi syndrome in the accelerated phase. Saudi Med J.

[17] Ghaffari J, Rezaee SA, Gharagozlou M (2013). Chédiak-Higashi syndrome. Journal of Pediatrics Review.

